# The Holistic Processing Account of Visual Expertise in Medical Image Perception: A Review

**DOI:** 10.3389/fpsyg.2017.01620

**Published:** 2017-09-28

**Authors:** Heather Sheridan, Eyal M. Reingold

**Affiliations:** ^1^Department of Psychology, University at Albany, State University of New York, Albany, NY, United States; ^2^Department of Psychology, University of Toronto, Mississauga, ON, Canada

**Keywords:** medical image perception, visual expertise, radiology, holistic processing, attention, time course, expert performance, eye movements

## Abstract

In the field of medical image perception, the holistic processing perspective contends that experts can rapidly extract global information about the image, which can be used to guide their subsequent search of the image (Swensson, 1980; Nodine and Kundel, 1987; Kundel et al., 2007). In this review, we discuss the empirical evidence supporting three different predictions that can be derived from the holistic processing perspective: Expertise in medical image perception is domain-specific, experts use parafoveal and/or peripheral vision to process large regions of the image in parallel, and experts benefit from a rapid initial glimpse of an image. In addition, we discuss a pivotal recent study (Litchfield and Donovan, 2016) that seems to contradict the assumption that experts benefit from a rapid initial glimpse of the image. To reconcile this finding with the existing literature, we suggest that global processing may serve multiple functions that extend beyond the initial glimpse of the image. Finally, we discuss future research directions, and we highlight the connections between the holistic processing account and similar theoretical perspectives and findings from other domains of visual expertise.

## Introduction

Human visual expertise in many fields reflects complex cognitive and perceptual processing that is developed over the course of many hours of practice and training (for reviews, see Ericsson et al., 1993, 2006; Reingold and Sheridan, 2011). In the domain of medical image perception, extensive training and experience is required to learn how to interpret medical visualizations, which are visual images that represent human anatomical structures or functions. As discussed by Krupinski (2010), the field of radiology has the largest volume of medical imaging exams, but medical image perception is a component of a wide range of other medical specialties, including cardiology, dermatology, radiation oncology, pathology, and ophthalmology. The task of interpreting medical images is often challenging; in the field of radiology, inter-rater variability is high and abnormalities are missed as often as 30% of the time (e.g., Birkelo et al., 1947; Guiss and Kuenstler, 1960; Austin et al., 1992; Bird et al., 1992). Furthermore, the volume and the complexity of the medical image perception task is increasing due to recent technological advances, such as the increased use of multislice imaging – including magnetic resonance imaging (MRI) and computed tomography (CT) – and the advent of telemedicine, which utilizes video images to enable medical decisions to be made from a distance.

Given the importance of the accurate interpretation of medical images to patient outcomes, there has been extensive work focused on understanding expertise differences during medical perception tasks. As summarized by previous reviews of this topic (Norman et al., 1992; Nodine et al., 1999; Krupinski, 2000b, 2010; Nodine and Mello-Thoms, 2000, 2010; Ericsson, 2004; Taylor, 2007; Atkins et al., 2008; Gegenfurtner et al., 2011, 2013, 2017; Reingold and Sheridan, 2011; Drew et al., 2013; van der Gijp et al., 2014, 2016; Wolfe et al., 2016; Blondon et al., 2015; Litchfield and Donovan, 2017), expertise in a wide range of medical image perception tasks entails both efficiency and accuracy; Relative to novices, experts typically require less time to make a decision while at the same time achieving higher levels of diagnostic accuracy (i.e., the ability to accurately detect the presence or absence of a disease or health condition, and/or to make effective decisions about patient care). In the field of medical image perception, diagnostic accuracy is typically measured using signal detection theory measures, including receiver operating characteristic (ROC) metrics (for further discussion, see Krupinski and Jiang, 2008; Chakraborty, 2010; Hillis, 2010; Tourassi, 2010).

In the current review, our main goal is to discuss empirical support for the view that expertise in medical image perception reflects the ability of experts to engage in holistic or global processing of overall patterns. This view (henceforth referred to as the *holistic processing account)* is encompassed by several different theoretical frameworks, including the holistic model (Kundel et al., 2007), the global-focal search model (Nodine and Kundel, 1987), and the two-state detection model (Swensson, 1980). Accordingly, in the review below, we begin by outlining the specific assumptions of the holistic processing account of expertise differences in medical image perception. Next, we briefly summarize the empirical evidence supporting holistic processing, with a focus on the key role of eye tracking methodology, which has emerged as a important tool for studying expert/novice differences in medical image perception tasks (for a review, see Reingold and Sheridan, 2011). In our summary of the literature, we do not attempt to duplicate our prior review (Reingold and Sheridan, 2011). Instead, we provide a brief summary of the literature with a focus on recent developments that are relevant to the holistic processing account of medical image perception. As described below, although there is a wide range of empirical support for the holistic processing view, the holistic processing advantage of experts was not supported by an innovative recent study (Litchfield and Donovan, 2016) that employed the flash-preview moving window paradigm (Castelhano and Henderson, 2007; Litchfield and Donovan, 2017). This paradigm allowed for a more direct test of the role of holistic processing by using gaze-contingent methodology to isolate the initial glimpse of the image from subsequent search behavior. Therefore, we conclude our summary of the literature by discussing how this recent study by Litchfield and Donovan (2016) could be reconciled with the prior body of work supporting the holistic processing view. Finally, we conclude by discussing future research directions that might further advance our understanding of the role of holistic processing during medical image perception.

## Theoretical Assumptions of the Holistic Processing Account

A variety of theoretical frameworks have incorporated “holistic” or “global” processing mechanisms as a core component of expertise in medical image perception, including the holistic model (Kundel et al., 2007), the global-focal search model (Nodine and Kundel, 1987), and the two-state detection model (Swensson, 1980). Moreover, several other conceptualizations of holistic processing were initially developed in other domains and later applied to the field of medical image perception, including the view that visual scenes are processed using two distinct pathways (Torralba et al., 2006; Wolfe et al., 2011; Drew et al., 2013), and chunking/template theory from the domain of chess (Chase and Simon, 1973a,b; Gobet and Simon, 1996, 2000; Wood, 1999). As discussed below, these different theoretical perspectives offer distinct – but partially overlapping – conceptualizations of holistic processing during medical image perception.

According to the global-focal search model (Nodine and Kundel, 1987; see also, Nodine and Mello-Thoms, 2000, 2010), medical experts rapidly extract a global impression of an image, and this impression consists of a comparison between the contents of the image, and the expert’s prior knowledge about the visual appearance of normal and abnormal medical images (i.e., the expert’s *schemas*). This global impression enables experts to identify perturbations, which are deviations from the expert’s schemas that indicate possible abnormalities. Using this global impression, medical experts can then direct their eyes toward the locations of possible abnormalities, so that these locations can be further examined using the *fovea* (i.e., the small region of the human eye that permits the extraction of detailed visual information). Although these global and focal processing stages are conceptualized as operating serially (such that the global impression of the image precedes focal processing), Nodine and Mello-Thoms (2000, p. 869) note that the global and focal processing stages can be recursive, such that after the completion of focal processing of a possible abnormality “attention shifts back to the medical image for a new global impression flagging another perturbed region, focal analysis searches it, a new object may be recognized and recursive testing for abnormalities continues until the observer is satisfied that enough evidence has accumulated to make a diagnostic decision.”

Similar to the global-focal search model, the two-stage detection model (Swensson, 1980) emphasizes the important role of holistic processing in medical image perception. According to the two-stage detection model, experts acquire perceptual mechanisms through extensive training, which serve as an initial filter that automatically identifies features that require further examination. These perceptual mechanisms are capable of filtering out normal anatomical structures, in order to rapidly direct the expert’s attention toward regions of the image that contain potential abnormalities. Thus, both the two-stage detection model and the global-focal search model assume that experts can process large regions of an image using their parafoveal and peripheral vision (i.e., regions of the visual field that are outside of the fovea), which enables them to rapidly identify potentially relevant regions of the images that can subsequently be examined further using foveal vision. As well, similar to the global-focal search model, the two-stage detection model adopts two serial stages of processing (although unlike the global-focal search model, these stages were not assumed to be recursive). More specifically, according to the two-stage detection model, the perceptual mechanisms comprising the initial filter (which is assumed to operate pre-attentively) provide input for a subsequent stage of processing. During this subsequent stage, attention is focused on potentially relevant specific features, and “Each selected feature receives an explicit evaluation by a cognitive process which determines whether (and at what level of confidence) that feature will be reported as a target” (Swensson, 1980, p. 11). Swensson (1980) used signal detection theory to implement these two stages (i.e., the initial “preattentive filter” and the subsequent “cognitive evaluation”) within a formal model in order to simulate the ROC curves obtained from empirical studies of the diagnostic performance of radiologists.

Congruent with the two-stage detection model and the global-focal search model, Kundel et al. (2007) contended that the development of expertise in medical image perception reflects a shift from a comparatively slow “search-to-find” mode to a more rapid holistic mode. The holistic mode involves a rapid global assessment of the image, which enables the expert to identify perturbations that could be potential abnormalities. The expert then subsequently initiates the “search to find” mode, which involves shifting their gaze to potentially relevant locations, as well as scanning the image to locate additional abnormalities that were not salient enough to be noticed during the initial global assessment. Kundel et al. (2007) also points out that global processing can operate in parallel with the search-to-find mode, so global information can continue to “flag” new abnormalities even after scanning is already in progress. Kundel et al. (2007) contends that the ability to engage in global processing during medical image perception requires extensive training and experience to develop. Thus, in contrast to experts, novices have not acquired the ability to engage in the rapid holistic mode, and are therefore primarily limited to discovering abnormalities using the slower search-to-find mode.

The holistic processing views described above also resemble several other theoretical perspectives that were initially developed outside of the domain of medical expertise. For example, as discussed by Drew et al. (2013), the above models of medical image perception are consistent with the view that visual scenes are processed using two distinct pathways (Torralba et al., 2006; Wolfe et al., 2011; Drew et al., 2013). More specifically, according to this view, two pathways are operating in parallel during scene perception tasks. A non-selective visual pathway permits the rapid extraction of statistical or global visual information from a wide field of view, and a selective visual pathway permits the extraction of detailed visual information to support object recognition. Similar to the above theoretical accounts, this viewpoint assumes that the non-selective pathway can rapidly draw attention to potentially relevant regions that can subsequently be examined in more detail using the selective visual pathway. The selective and non-selective pathways are analogous to the global and focal stages proposed by the above theories. Moreover, the selective and non-selective pathways are assumed to operate in parallel, which echoes Kundel et al. (2007)’s assumption that global and focal processing can operate in parallel, but contrasts with Swensson (1980)’s assumption of two serial stages.

In addition to the above perspectives, as pointed out by (Wood, 1999), the holistic processing component of medical expertise might reflect similar mechanisms as the concept of *chunking* (Miller, 1956) that was postulated to be a central component of the remarkable proficiency shown by chess experts relative to novices (for a review, see Reingold and Sheridan, 2011). Specifically, Chase and Simon (1973a,b) hypothesized that chess experts acquire memory representations for chunks of chess-related visual information (e.g., groups of chess pieces), which are supplemented by larger memory structures called templates (Gobet and Simon, 1996, 2000). According to chunking and template theory, chunks and templates are memory structures that are acquired over the course of many hours of practice, and these memory structures facilitate performance by allowing experts to rapidly encode chess configurations in terms of larger patterns. Thus, chunking and template theory postulates that a key component of visual expertise is the ability to process domain-related visual stimuli in terms of larger patterns, rather than individual features. This ability of experts to process larger patterns via chunking leads to the prediction that medical experts can process visual information in parallel across a wide region of the image during a given eye fixation, resulting in a larger visual span for medical experts than novices (note that the term visual span is also referred to in the literature as the perceptual span or the span of effective vision, see Jacobs, 1986; Rayner, 1998).

Thus, a variety of theoretical perspectives have hypothesized that holistic (or global) processing is a core component of expertise in medical image perception. Despite the fact that the conceptualizations of holistic processing in this field of medical expertise are somewhat vague and non-uniform, in the present review we explored the empirical support for three predictions that constitute points of overlap across the holistic processing accounts outlined above: (1) Medical expertise involves a domain-specific perceptual component, (2) Medical experts use their parafoveal and/or peripheral vision to process large regions of an image in parallel, and (3) Medical experts benefit from a rapid initial “glimpse” of an image.

Moreover, while exploring the above predictions, we emphasize the key role of eye tracking methodology in testing the holistic processing account (see Reingold and Sheridan, 2011). Since foveal vision is required to process complex medical images, eye movements are necessary to align the fovea with regions of interest. Eye movements are therefore an essential component of the medical imaging task, and monitoring eye movements can reveal insights about ongoing processing and the allocation of attention, without requiring additional responses or reliance on self-reports. Moreover, as reviewed by Reingold and Sheridan (2011), eye movements can reveal information that experts are not aware of and/or unable to verbalize.

## Prediction #1: Medical Expertise Involves A Domain-Specific Perceptual Component

A key point of overlap across the various models of holistic processing is the assumption that holistic processing reflects a domain-specific perceptual component. Specifically, while not ruling out the important contributions of cognitive and higher level decision-making processes, the various holistic models have proposed that medical image perception (at least in part) reflects a variety of (non-mutually exclusive) perceptual mechanisms, such as the ability to use schemas of the appearance of human anatomy to recognize departures from normal tissue (i.e., “perturbations”; see e.g., Nodine and Kundel, 1987; Nodine and Mello-Thoms, 2000, 2010), the ability to encode visual features as larger perceptual units called chunks or templates (Wood, 1999), and the ability to rapidly encode a “gestalt” of a scene which allows the extraction of a rapid understanding of the scene using global or statistical information (Drew et al., 2013). These conceptualizations of holistic processing share the prediction that medical image perception reflects domain-specific perceptual abilities that are acquired through extensive training.

A wide range of findings have supported the prediction that visual expertise in medicine is domain specific, which is in line with similar findings from other domains such as chess (for a review, see Reingold and Sheridan, 2011). Although expert radiologists performed better than novices at medical visual discrimination tasks (Snowden et al., 2000), expertise differences were not found for a variety of control tasks involving visual search and/or memory tasks with non-medical images (Nodine and Kundel, 1997; Nodine and Krupinski, 1998; Moise et al., 2005; Evans et al., 2011; Evans et al., 2016b; Litchfield and Donovan, 2016). For example, radiologists did not perform better than novices when tested with control visual search tasks that involved searching for the character WALDO and searching for the word NINA (Nodine and Kundel, 1997; Nodine and Krupinski, 1998), radiologists and laypersons showed a similar pattern of results on a comparative visual search task that closely resembled medical imaging tasks (Moise et al., 2005), radiologists and medical students were equally able to detect repetitions on an n-back task involving images of faces, rooms and tools (Bilalić et al., 2016), medical experts and naïve observers showed equal performance on a recognition memory task involving visual stimuli (scenes and objects) from outside of the experts’ domain of expertise (Evans et al., 2011; for similar findings, see Myles-Worsley et al., 1988; Evans et al., 2016b). Moreover, when the same observers were assessed at multiple points in their training, visual search patterns changed over time, which suggests that expertise in medical image perception is acquired gradually as observers gain experience with domain-specific visual patterns (Krupinski and Weinstein, 2011; Krupinski et al., 2013).

Taken together, these results suggest that the perceptual advantages of medical experts are domain specific. Moreover, Nodine and Mello-Thoms (2010, p. 139) note that medical expertise is not only domain specific, but also subdomain specific, such that “acquiring expertise in interpreting chest radiographs does not directly transfer to interpreting mammograms.” Nodine and Mello-Thoms (2010, p. 139) note that sub-domain specificity might be advantageous because “Limiting knowledge to a specific standardized anatomic scene may facilitate tuning of specific perceptual and cognitive skills that give the expert a distinct advantage.” Thus, although further work is needed to clarify the specific mechanisms that contribute to holistic processing, there is strong evidence in support of the holistic account’s assumption that domain-specific perceptual mechanisms are a core component of expertise in medical image perception.

## Prediction #2: Medical Experts Use Their Parafoveal And/Or Peripheral Vision To Process Large Regions of An Image In Parallel

Another common thread across the different accounts of holistic processing is the idea that experts can process large regions of the image in parallel using their parafoveal and/or peripheral vision. As described below, this prediction has been supported by a wide range of empirical findings, including findings that expert performance is disrupted by manipulations that obstruct their ability to process large regions of the image, findings that experts can rapidly detect abnormalities located outside of their fovea, and the remarkable efficiency of experts as indexed by a variety of eye tracking measures (see **Table 1**).

**Table 1:** Experts have more efficient searches than novices, as indexed by a variety of eye tracking measures (see text for details).

Dependent variable	Expertise findings and references
Total viewing times	Less time spent processing each image as expertise increased (Krupinski, 1996a,b, 2000a; Manning et al., 2003, 2006; Krupinski et al., 2006; Alzubaidi et al., 2009; Wood et al., 2013; Brunyé et al., 2014; Giovinco et al., 2015; Assaf et al., 2016; Kok et al., 2016)
Number of saccades/ fixations	Fewer fixations/saccades as expertise increased (Manning et al., 2003, 2006; Krupinski et al., 2006, 2013; Alzubaidi et al., 2009; Donovon and Litchfield, 2013; Voisin et al., 2013; Brunyé et al., 2014; Assaf et al., 2016)
Saccade length	Longer saccades as expertise increased (Manning et al., 2003, 2006; Krupinski et al., 2006, 2013; Kundel et al., 2007; Kok et al., 2012; Assaf et al., 2016; but see Kundel and Nodine, 2004)
Scanpath length	Shorter scanpaths as expertise increased (Kundel and La Follette, 1972; Krupinski, 1996a,b, 2000a; Krupinski and Borah, 2006)
Time to first fixation on abnormality (search latency)	Reduced time to first fixation on the abnormality as expertise increased (Krupinski, 1996a,b, 2000a, 2005; Nodine et al., 1996a,b; Nodine and Kundel, 1997; Kundel and Nodine, 2004; Kundel et al., 2007; Wood et al., 2013; but see Donovon and Litchfield, 2013)
Proportion of time fixating relevant regions	Increased percentage of time fixating relevant regions (i.e., regions containing abnormalities) as expertise increased (Donovon and Litchfield, 2013; Wood et al., 2013)
Dwell times	Shorter dwell times as expertise increased (Donovon and Litchfield, 2013; Krupinski et al., 2013; Voisin et al., 2013) Longer dwell times on lesions as expertise increased (Krupinski, 1996a,b, 2000b; Nodine et al., 1996a,b, 2002; Kundel and Nodine, 2004; but see Krupinski, 2005)
Fixation times	Shorter fixations as expertise increased (Alzubaidi et al., 2009; Kok et al., 2012; Assaf et al., 2016; but see Giovinco et al., 2015) Increased fixation rate (number of fixations per second) as expertise increased (Giovinco et al., 2015)

One of the most direct approaches to testing the assumption that experts are utilizing parafoveal/peripheral processing is to examine the impact of visual manipulations that prevent experts from viewing large regions of an image. For example, (Carmody et al., 1980b) reported superior accuracy by radiologists under viewing conditions that revealed the full image, relative to a segmented viewing condition that divided the image into six sections that were presented one at a time (for similar findings, see Carmody, 1984). Similarly, Swensson et al. (1982, 1985) reported higher accuracy under free viewing conditions, relative to a condition in which radiologists were instructed to focus on particular regions and features. Gaze-contingent window paradigms have also revealed better performance when larger regions of the image are visible, relative to conditions that restricted visibility to a smaller portion of the image (Kundel et al., 1984, 1991). Moreover, in the field of pathology, when observers were permitted to choose their level of magnification, the experts spent a greater proportion of time at low magnification (i.e., the level of magnification that provided a more “global” view of the image), compared to both intermediates and novices (Jaarsma et al., 2015; for related findings, see Jaarsma et al., 2016). Taken together, these findings support the holistic processing view by revealing that experts benefit from conditions that permit the processing of overall patterns.

Moreover, another source of evidence supporting the key role of parafoveal/peripheral processing is the remarkable ability of medical experts to extract relevant information from a briefly presented image. For example, expert radiologists were able to detect 70% of abnormalities when chest films were displayed for only 200 ms (Kundel and Nodine, 1975; for similar findings see Kundel and Nodine, 1975; Carmody et al., 1980a, 1981; Oestmann et al., 1988; Gale et al., 1990; Mugglestone et al., 1995; Evans et al., 2013, 2016a; Jaarsma et al., 2014; Houghton et al., 2015), and experts were capable of detecting some nodules that were 15° away from their point of fixation (Carmody et al., 1980a). Importantly, the brief exposure conditions in these studies do not provide sufficient time for the radiologists to move their eyes to the locations of the abnormalities, thereby ruling out alternative explanations of the results (such as the notion that medical expertise solely reflects foveal processing).

Complimenting the brief exposure studies, measurements of the “time to decision” (i.e., the time between the image onset to lesion detection) revealed that expert mammographers can rapidly report abnormalities; Specifically, as reviewed by Nodine and Mello-Thoms (2010), experts were able to detect 71% of lesions within 25 s, but novices required 40 s to detect 46% of abnormalities. Moreover, the reporting times of experts revealed a rapid reporting phase followed by more gradual reporting, whereas less experienced observers revealed a more constant rate of reporting abnormalities (Christensen et al., 1981; Nodine et al., 2002). Taken together with the brief exposure studies, this pattern of results suggests that experts could have been using their peripheral and parafoveal vision to facilitate their rapid detection of abnormalities.

Building on the above paradigms, prior work has also utilized eye tracking methodology to obtain a wide range of measures of scanpath efficiency. As discussed below, the scanpaths of medical experts are extremely efficient, as exemplified by reports that experts only processed an average of 15–20% of the image with foveal vision (for a review, see Nodine and Mello-Thoms, 2010). While these findings are consistent with the idea that experts are using their parafoveal/peripheral vision to guide their search, it’s also possible that experts are guiding their search using other mechanisms, such as their greater medical knowledge about the likely location of potential abnormalities. Moreover, as discussed by Litchfield and Donovan (2016), after scanning of an image is underway (i.e., after enough time has elapsed to permit eye movements), it becomes difficult to disentangle the relative contributions of foveal vs. parafoveal/peripheral processing. While not definitive evidence, the efficient scanpaths of experts are nevertheless consistent with the holistic processing account’s assumption that parafoveal/peripheral processing is an important component of medical image perception.

In an early investigation of the scanpath patterns of medical experts, Tuddenham and Calvert (1961) asked radiologists to use a spotlight to examine roentgenograms of both normal and abnormal chests in a dimly lighted room. By recording the movements of the spotlight (using a motion-picture camera), it was possible to infer the scanning patterns of the radiologists, which were revealed to be highly variable across different observers. As summarized in **Table 1**, subsequent work revealed a wide range of differences in scanpath characteristics as a function of expertise (Reingold and Sheridan, 2011). Relative to novices, experts displayed shorter scan paths (Kundel and La Follette, 1972; Krupinski, 1996a,b, 2000a; Krupinski and Borah, 2006) and reduced total viewing times (Krupinski, 1996a,b, 2000a; Manning et al., 2003, 2006; Krupinski et al., 2006; Alzubaidi et al., 2009; Wood et al., 2013; Brunyé et al., 2014; Giovinco et al., 2015; Assaf et al., 2016; Kok et al., 2016). Furthermore, relative to novices, the scan paths of experts are characterized by larger saccades (Manning et al., 2003, 2006; Krupinski et al., 2006, 2013; Kundel et al., 2007; Kok et al., 2012; Assaf et al., 2016), fewer numbers of fixations/saccades (Manning et al., 2003, 2006; Krupinski et al., 2006, 2013; Alzubaidi et al., 2009; Donovon and Litchfield, 2013; Voisin et al., 2013; Brunyé et al., 2014; Assaf et al., 2016), less coverage of the image (Krupinski, 1996a,b, 2000a; Manning et al., 2003, 2004, 2006; Kok et al., 2016), a greater proportion of time spent fixating on relevant regions and/or regions containing abnormalities (Donovon and Litchfield, 2013; Wood et al., 2013), and greater consistency in their scan paths (Hu et al., 1994; Mello-Thoms et al., 2002; Mello-Thoms, 2003; Leong et al., 2007; Kok et al., 2016). Moreover, less experienced observers focus their attention on visually salient regions, whereas experts focus on regions that are relevant but not necessarily visually salient (Kundel and La Follette, 1972; Matsumoto et al., 2011; Brunyé et al., 2014) and a spatial frequency analysis indicated that less experienced observers’ search strategies are influenced by the local saliency of the lesions to a greater extent than experts (Mello-Thoms et al., 2003). Finally, as further evidence that the scanpaths of experts are highly systematic, radiologists were substantially more efficient in their search patterns in comparison to scanpaths that were generated by a random walk algorithm (Nodine and Kundel, 1987; For related findings, see Geisler and Najemnik, 2005).

Building on the above findings, in the field of pathology, observers were shown a low magnification image and then asked to determine the locations that they would zoom into if they were to continue to examine an image (Krupinski et al., 2006, 2013). The expert pathologists were more likely to not fixate at all on their preferred zoom locations (Krupinski et al., 2006), which could signify a greater ability to process the image using their peripheral and parafoveal vision.

Consistent with the holistic processing account, efficient and consistent scanpaths imply that experts are using their parafoveal/peripheral vision to obtain a global impression of the image that enables them to rapidly move their eyes toward regions containing an abnormality. In particular, a key measure of the efficiency of the experts’ scanpath is the amount of time between the presentation of the image and the first eye fixation on a region containing an abnormality (i.e., *Time to First Fixation*). The time to first fixation is shorter for experts than novices (Krupinski, 1996a,b, 2000a, 2005; Nodine et al., 1996a,b; Nodine and Kundel, 1997; Kundel and Nodine, 2004; Kundel et al., 2007; Wood et al., 2013; but see also Donovon and Litchfield, 2013), and in some cases the experts could rapidly fixate on abnormalities in less than a second (Kundel et al., 2008). In contrast, a study by Donovon and Litchfield (2013) did not show significant expertise effects on time to first fixation, although they did show a numerical trend toward faster time to fixations as a function of expertise. In interpreting this finding, Donovon and Litchfield (2013) point out that the lack of significance may have been due to low power (a key methodological challenge in this literature is that it is often difficult to obtain a large sample of medical experts). Also, the x-ray inspection task from Donovon and Litchfield (2013)’s study included more subtle abnormalities than prior studies in the field of mammography, so stimulus and/or task differences are another potential reason for the conflicting pattern of results. As pointed out by Donovon and Litchfield (2013), the time to fixate on an abnormality tends to be longer for images with subtle or less conspicuous abnormalities, so the subtlety of the abnormality could be a possible boundary condition for showing rapid times to first fixations. Nevertheless, as reviewed by Gegenfurtner et al. (2011), the time to first fixation measure generally does decrease as a function of expertise, which is consistent with the holistic processing account’s prediction that experts are using their peripheral and parafoveal vision to process large areas of the image simultaneously.

## Prediction #3: Medical Experts Benefit From A Rapid Initial “Glimpse” of An Image

The different variants of the holistic processing account share the prediction that experts can rapidly extract diagnostically relevant information from their initial glimpse of an image. As noted by Drew et al. (2013, p. 265), “An important implication of the existence of a non-selective pathway is that even the briefest of glances at an image may contain valuable information that might be exploited in the development of teaching tools or more effective computer-aided detection algorithms.” Similarly, Kundel et al. (2007) emphasized the importance of the initial glance in stating: “The best observers actually jumped directly to the cancer on first seeing the image. These data support the hypothesis that an initial global image analysis produces a holistic perception that enables the rapid identification of abnormalities…” (Kundel et al., 2007, p. 401).

Empirical support for the importance of the initial glimpse includes the previously mentioned findings that experts can rapidly detect abnormalities in briefly presented images (Kundel and Nodine, 1975; for similar findings see Carmody et al., 1980a, 1981; Oestmann et al., 1988; Gale et al., 1990; Mugglestone et al., 1995), as well as the findings that experts display efficient scanpaths with rapid times to the first fixation on the abnormality (Krupinski, 1996a,b, 2000a, 2005; Nodine et al., 1996a,b; Nodine and Kundel, 1997; Kundel and Nodine, 2004; Kundel et al., 2007, 2008; Donovon and Litchfield, 2013; Wood et al., 2013). Of course, there are limits to the amount of information that can be extracted from the expert’s initial glance at an image, as shown by the finding that experts were at chance levels when asked to localize the abnormality under brief exposure conditions, even though they could detect the presence or absence of an abnormality at above chance levels (Evans et al., 2013), findings that subtle or less conspicuous abnormalities require more time and/or foveal processing to be detected (e.g., Carmody et al., 1981; Oestmann et al., 1988) findings that diagnostic accuracy decreases as distance from the fovea increases (Carmody et al., 1980a), and findings that diagnostic accuracy is substantially higher under conditions that permit longer viewing times relative to brief exposure conditions (e.g., Kundel and Nodine, 1975; Oestmann et al., 1988; Mugglestone et al., 1995; Houghton et al., 2015).

An additional method for studying the role of the initial glance is to explore the time course of processing over the course of a trial. As previously mentioned, measurements of the “time to decision” (i.e., the time between the image onset to lesion detection) revealed that experts show a rapid reporting phase followed by more gradual reporting, whereas less experienced observers revealed a more constant rate of reporting abnormalities (Christensen et al., 1981; Nodine et al., 2002). Moreover, to study the time course of processing across a trial, Nodine and Kundel (1987) observed that radiologists displayed short fixations (100–200 ms) followed by longer fixations (>600 ms), and concluded that “this sequence of alternating between globally surveying the image and following up with in-depth examination of distinctive anatomical detail characterizes a fundamental perceptual-cognitive strategy behind skilled search.” Although these findings seem to support the notion of two sequential stages of processing (e.g., global and focal processing), there are several alternative explanations. With respect to the “time to decision” findings, it is possible that the rapid reporting phase could reflect the detection of more obvious or conspicuous abnormalities (which can be rapidly detected), followed by the reporting of more subtle abnormalities (for a related discussion, see Donovon and Litchfield, 2013). Moreover, with respect to the fixation duration findings, it is possible that the shorter initial fixations reflected perceptual encoding whereas the later fixations reflected decision making (for further discussion, see Glaholt and Reingold, 2011; see also Reingold and Charness, 2005, for a similar pattern of results in the domain of chess).

Building on the above findings, a pivotal recent study by Litchfield and Donovan (2016) was designed to more directly test the holistic processing account’s prediction that the initial glance contains diagnostically relevant information. Specifically, to differentiate between the initial “glimpse” of the image and subsequent processing, Litchfield and Donovan (2016) used a gaze-contingent paradigm called the *flash-preview moving window (FPMW)* paradigm (Castelhano and Henderson, 2007; Litchfield and Donovan, 2017). Using this paradigm, observers were first shown a brief preview of a medical image (for 250-ms), and they were then asked to search the same image for an abnormality under conditions that restricted their vision to a small gaze-contingent moving window (with a radius of 2.5°).

Given the holistic processing account’s assumption that experts rapidly gather global information during their initial glimpse of an image, Litchfield and Donovan (2016) predicted better diagnostic performance following the brief preview, relative to a condition that presented a mask instead of a preview. Unexpectedly, for both experts and novices, they did not find a benefit of the preview for diagnostic accuracy, and in some cases diagnostic performance was worse for the preview relative to the mask condition. The brief preview also largely did not impact search efficiency, with the exception that the experts (but not the novices) showed a non-significant trend toward a preview benefit for search efficiency (as indexed by a reduced number of fixations and reduced time to the first fixation on the abnormality), but this small difference only occurred when observers were examining a single type of image across trials (i.e., chest x-rays), and not when they were asked to examine a variety of different types of medical images across trials (i.e., chest x-rays, brain images, and skeletal images).

Litchfield and Donovan (2016)’s findings contradict the assumption that holistic processing is isolated to the initial glance at an image during medical image perception. However, although some models have adopted this rigid assumption that holistic processing only occurs during the initial stage of processing an image (Swensson, 1980), other models have instead assumed that holistic processing can continue throughout the trial, by operating either in parallel (Kundel et al., 2007; Drew et al., 2013) or recursively (Nodine and Mello-Thoms, 2000) with other types of processing. For example, Drew et al. (2013) assumed that the selective and non-selective visual pathways are operating in parallel, Kundel et al. (2007, p. 397) assumed that “Global retinal analysis and focal feature analysis are simultaneously active during the fixations…”, Nodine and Mello-Thoms (2000) assumed that the global and focal stages of processing could be recursive, and Kundel (2000, p. 846) stated that the “…two different types of scene analysis, global and focal, are performed sequentially, sometimes within a single fixation and sometimes in a cluster of fixations centered on a particular location in the image.”

Critically, by assuming that holistic processing is ongoing throughout the trial instead of isolated to the initial glimpse, it’s possible to reconcile the holistic processing account with Litchfield and Donovan (2016)’s findings. Specifically, one possible explanation for Litchfield and Donovan (2016)’s findings is that the small size of the gaze-contingent window interfered with the ongoing global processing that occurs during standard free-viewing conditions. This ongoing global processing may serve important functions during medical image perception tasks, such as the facilitation of visual comparisons between foveated features and the surrounding context. In fact, in a study by Carmody et al. (1984), experts claimed to be making visual comparisons even though comparison saccades were not evident, which may suggest that experts are using global processing to make visual comparisons without moving their eyes. It is possible that these visual comparisons are particularly important for the difficult task of distinguishing between visually similar distractors and true abnormalities. Litchfield and Donovan (2016) point out that the preview may have drawn attention to distracting features that resembled abnormalities, and we speculate that the presence of the window during scanning impaired global visual comparisons that could otherwise have enabled observers to rule out these distractors. Moreover, to compensate for the lack of ongoing global processing in the moving window condition, the observers could have attempted to remember their initial glimpse of the image, and an imprecise or degraded memory of the initial glimpse could have impaired their performance. As discussed below, future research can continue to investigate global processing mechanisms to further clarify the intriguing findings reported by Litchfield and Donovan (2016).

## Summary and Future Research Directions

In support of the holistic processing account of medical image perception, the present review discussed findings that medical expertise involves a domain-specific perceptual component, and findings that experts use their parafoveal and/or peripheral vision to process large regions of an image in parallel. Moreover, we highlighted recent findings by Litchfield and Donovan (2016) that contradict the idea that holistic processing is limited to the initial glance at the image, which we suggested was consistent with a theoretical shift in the literature toward conceptualizing holistic processing as an ongoing process that continues to play an important role even after the initial “glimpse” of the image.

Building on these results, the predictions of the holistic processing account could be further tested using Reingold et al. (2001)’s approach of systematically varying the size of the gaze contingent window to precisely quantify the size of the visual span of observers as a function of expertise. To the extent that the size of the visual span increases as a function of expertise, this paradigm would provide strong support for the holistic processing account’s prediction that expertise in medical image perception is associated with the ability to process large areas of the image using parafoveal/peripheral vision. Moreover, to the extent that expertise differences in the size of the visual span are limited to domain-specific visual stimuli, this paradigm could also provide support for the holistic processing account’s prediction that expertise in medical image perception is domain-specific. Such a pattern of results would replicate Reingold et al. (2001)’s findings that chess experts display a larger visual span than less skilled players while viewing domain-related stimuli (i.e., configurations from chess games), but not while viewing random configurations of chess pieces. Moreover, this approach of measuring visual span could be used to explore the impact of a variety of variables on parafoveal/peripheral processing during medical image perception, including different viewing conditions, image types, the subtlety of the abnormalities, and the point in time in the process of interpreting an image.

As well, it will be important to test the assumptions of the holistic processing account in a wider range of imaging modalities and tasks, in light of the ongoing trend toward greater complexity and volume, and more dynamic images instead of static images. Interestingly, it is possible that some of the key findings reviewed above may not necessarily extend to other image methodologies and tasks; For example, experts displayed shorter saccades than novices during a CT task, which may have been indicative of their use of a “drilling” strategy that focused their attention on a specific location in space (Bertram et al., 2013; For a review of eye tracking findings in volumetric imaging, see Venjakob and Mello-Thoms, 2015).

Finally, future work could further explore the extent to which holistic processing in medicine might reflect similar mechanisms as other conceptualizations of holistic processing in domains such as scene perception (Torralba et al., 2006; Castelhano and Henderson, 2007; Wolfe et al., 2011), face perception (for reviews, see Piepers and Robbins, 2012; Tanaka and Gordon, 2012) and chess expertise (for a review, see Reingold and Sheridan, 2011). Toward this goal, neuroimaging studies have uncovered common neural substrates in radiological expertise and other domains of visual expertise (Bilalić et al., 2016; for related findings, see Harley et al., 2009; Melo et al., 2011). In particular, Bilalić et al. (2016) revealed expertise differences in brain activation in the fusiform face area (FFA), which was previously linked to the holistic processing of faces (Kanwisher et al., 1997), as well as other visual stimuli (Gauthier et al., 2000). In interpreting their results, Bilalić et al. (2016) concluded that the FFA’s sensitivity to X-rays suggests that radiological expertise reflects holistic processing. Future work could continue to make connections across domains of expertise, with the goal of further clarifying the nature of holistic processing during medical image perception.

## Author Contributions

Conception and design of review (HS and ER), writing and revising review (HS and ER).

## Conflict of Interest Statement

The authors declare that the research was conducted in the absence of any commercial or financial relationships that could be construed as a potential conflict of interest.
